# Genomic and Epigenomic Profile of Uterine Smooth Muscle Tumors of Uncertain Malignant Potential (STUMPs) Revealed Similarities and Differences with Leiomyomas and Leiomyosarcomas

**DOI:** 10.3390/ijms22041580

**Published:** 2021-02-04

**Authors:** Donatella Conconi, Serena Redaelli, Andrea Alberto Lissoni, Chiara Cilibrasi, Patrizia Perego, Eugenio Gautiero, Elena Sala, Mariachiara Paderno, Leda Dalprà, Fabio Landoni, Marialuisa Lavitrano, Gaia Roversi, Angela Bentivegna

**Affiliations:** 1 School of Medicine and Surgery, University of Milano-Bicocca, 20900 Monza, Italy; serena.redaelli@unimib.it (S.R.); andreaalberto.lissoni@unimib.it (A.A.L.); m.paderno@campus.unimib.it (M.P.); leda.dalpra@unimib.it (L.D.); fabio.landoni@unimib.it (F.L.); marialuisa.lavitrano@unimib.it (M.L.); gaia.roversi@unimib.it (G.R.); 2 Clinic of Obstetrics and Gynecology, San Gerardo Hospital, 20900 Monza, Italy; 3 Department of Biochemistry and Biomedicine, School of Life Sciences, University of Sussex, Falmer, Brighton BN1 9RH, UK; cc677@sussex.ac.uk; 4 Division of Pathology, San Gerardo Hospital, 20900 Monza, Italy; patrizia.perego@asst-monza.it; 5 Medical Genetics Laboratory, San Gerardo Hospital, 20900 Monza, Italy; e.gautiero@asst-monza.it (E.G.); elena.sala@asst-monza.it (E.S.)

**Keywords:** uterine STUMP, array-CGH, copy number alterations, Genome Wide DNA Methylation

## Abstract

Uterine smooth muscle tumors of uncertain malignant potential (STUMPs) represent a heterogeneous group of tumors that cannot be histologically diagnosed as unequivocally benign or malignant. For this reason, many authors are working to obtain a better definition of diagnostic and prognostic criteria. In this work, we analyzed the genomic and epigenomic profile of uterine smooth muscle tumors (USMTs) in order to find similarities and differences between STUMPs, leiomyosarcomas (LMSs) and leiomyomas (LMs), and possibly identify prognostic factors in this group of tumors. Array-CGH data on 23 USMTs demonstrated the presence of a more similar genomic profile between STUMPs and LMSs. Some genes, such as *PRKDC* and *PUM2*, with a potential prognostic value, were never previously associated with STUMP. The methylation data appears to be very promising, especially with regards to the divergent profile found in the sample that relapsed, characterized by an overall CGI hypomethylation. Finally, the Gene Ontology analysis highlighted some cancer genes that could play a pivotal role in the unexpected aggressive behavior that can be found in some of these tumors. These genes could prove to be prognostic markers in the future.

## 1. Introduction

According to the 2014 WHO, uterine smooth muscle tumors (USMTs) consist of a group of histologically heterogeneous and clinically diverse diseases ranging from malignant leiomyosarcoma (LMS) to benign leiomyoma (LM). In between the aforementioned, lies a heterogeneous group of rare mesenchymal tumors that cannot be clearly categorized as benign or malignant lesions. Uterine smooth muscle tumors of uncertain malignant potential (STUMPs) do not meet benign variant or true malignancy criteria, due to their variability in histologic appearance, immunohistochemical profile and clinical outcome [1]. The unusual combination of findings generally renders STUMP diagnosis very difficult, while the different criteria adopted by pathologists affect its accuracy [2].

Although the majority of patients with uterine STUMPs have good outcomes, unexpected aggressive behavior can be found in some of the tumors [2]. Because of the neoplasm’s rarity, the etiology, prognostic factors, clinical outcomes and recurrence risks of these tumors are poorly defined [3]. Recently, a new classification of lesions according to genomic complexity has been described [4].

In this work, we analyzed the genomic profile of 23 USMTs by array-comparative genomic hybridization (array-CGH). In four samples the methylation profile was also investigated in order to find similarities and differences between STUMPs, LMSs and LMs, and possibly identify prognostic factors in this group of tumors.

## 2. Results

### 2.1. Pathologic Features

We performed an array-CGH analysis on 23 USMTs: 3 LMs, 14 STUMPs, 5 LMSs and 1 undifferentiated sarcoma (US), derived from a total of 20 patients who underwent first surgery between 1996 and 2013. The histological characteristics, the age at diagnosis and the type of surgery are listed in Table 1 (see also the Materials and Methods section). The median age at first diagnosis was 45.4 years old (ranging from 23 to 68).

Among the 14 STUMPs, 11/14 were primary tumors, of which four (STUMP1, STUMP3, STUMP4 and STUMP5) subsequently relapsed in either another STUMP or undifferentiated sarcoma (US) or LMS. For three patients it was possible to analyze both the primary STUMP and its recurrence (patients 4, 5 and 6). Primary STUMPs of patients 8 and 9 and relapsed tumor of patient 7 were not available. Among the LMSs, three were primary (LMS2, LMS3 and LMS4), while two were relapsed tumors (LMS1 and LMS5).

### 2.2. Array-CGH Results

STUMP samples revealed a wide range of copy number alterations (CNAs), from 17 to 365, with a median value of 86.5 (Table 2).

Two samples (STUMP3 and STUMP4) showed an exceptional number of CNAs (365 and 256 respectively). LMSs’ total number of CNAs ranged from 126 to 262, with a median value of 199. The only US sample analyzed showed the least number of CNAs compared to other sarcomas. Finally, LMs displayed an unexpected number of CNAs, ranging from 110 to 268, with a median value of 123. No statistically significant differences for the total number of CNAs were observed among the groups. A CNAs plot by chromosome for each patient is displayed in Figure S1.

**Figure 1:**
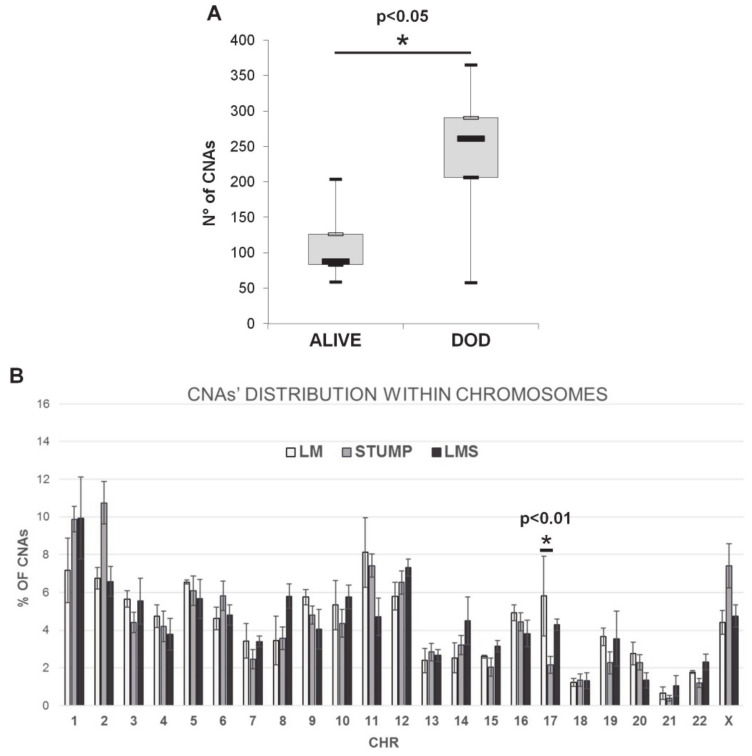
Array-CGH results. (**A**) Number of CNAs and follow-up status (Student *t* test *p* < 0.05). (**B**) CNAs’ distribution within chromosomes in leiomyomas, STUMPs and leiomyosarcomas (LM vs. STUMPs, ANOVA and Tukey test *p* = 0.0004). * statistically significant difference; - comparison between LM and STUMP

Interestingly, combining these data with the follow-up status, we found that primary STUMPs and primary LMS derived from patients with a worst prognosis (dead of disease, DOD) displayed a higher number of CNAs compared to patients’ samples with a good prognosis (alive with or without disease) (Figure 1A). As shown in Table 2, all patients with no evidence of disease (NED) had a longer disease.

ANOVA analysis performed on CNAs’ distribution within chromosomes in LMs, STUMPs and LMSs revealed a statistically significant difference only in chromosome 17 (LMs vs. STUMPs, Tukey test *p* = 0.0004; Figure 1B). This difference was also retained after splitting STUMPs in two different groups based on their ability to relapse (LMs vs. relapsing STUMPs, Tukey test *p* = 0.01; LMs vs. not relapsing STUMPs, Tukey test *p* = 0.0008, data not shown). No other differences were observed between the two groups of STUMPs or between STUMPs and LMSs.

In order to decipher a possible common code between STUMPs and LMSs or between STUMPs and LMs, we further searched among the numerous CNAs detected for those shared among STUMPs and LMSs, or STUMPs and LMs (at least three samples, Table S1). CNAs common to all histotypes were excluded because they were not useful for setting the STUMP genomic profile (data not shown). For the same reason, CNAs shared only among STUMPs were included in the analysis.

Curiously, losses represented a little percentage of the observed CNAs (6.2%). The majority was exclusive; few were shared between two samples. Moreover, known cancer-associated genes were not found (data not shown).

Despite the high number of CNAs, only seven were shared by LMs and STUMPs. Conversely, STUMPs and LMSs shared 47 CNAs, in particular gain/amplification of the regions containing cancer related genes as *PRKDC* (8q11.21; eight samples), *MLL3* (7q36.1; seven samples), *ROCK2* (2p25.1; six samples), *CTSB* (8p23.1; six samples) and *STAT2* (12q13.2; five samples) (Table S1 and Table 3). Interestingly, three out of four relapsing STUMPs shared an amplification of chromosome 2p24.1 including *PUM2* gene, which was not identified in other samples.

Narrowing down the analysis to the relapsing STUMPs and LMSs, we found some shared CNAs, especially in STUMP4 (the only relapsed in a LMS), including gain/amplification of the regions encompassing *STAG1* (3q22.2–22.3; four samples), *RFC4* (3q27.3; four samples), *CCRN4L* and *ELF2* (4q31.1; three samples) (Table 3).

As the amplification of the *BCL2* gene in primitive STUMPs and their corresponding relapsed tumors have been previously reported [5], an analysis of *BCL2* data was performed, revealing copy number gain or amplification in 13 samples: one leiomyoma (LM2), four primary STUMPs (STUMP10, STUMP11, STUMP12, STUMP13), two relapsing STUMPs (STUMP3 and STUMP4), one relapsed STUMP (STUMP6), three primary leiomyosarcomas (LMS2, LMS3 and LMS4) and two relapsed LMSs (LMS1 and LMS5) (Table S1 and Table 3).

### 2.3. Promoter CpG Island Methylation

**Figure 2:**
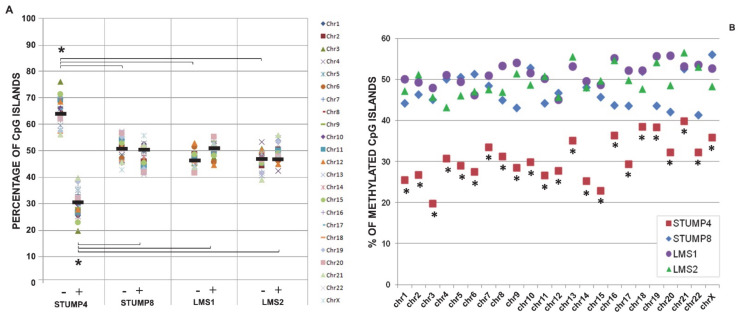
Methylation results. (**A**) Frequency of unmethylated (−) and methylated (+) CGIs for each sample. The methylation status for each chromosome is reported. Mean values are indicated with black dashes. STUMP4 vs. other samples * *p* < 0.01, Student’s *t* test. (**B**) Frequency of methylated CGI islands for each chromosome in each sample. STUMP4 vs. other samples * *p* < 0.01, Chi-square test.

We evaluated the CpG island methylation status of four samples, two STUMPs (STUMP4, STUMP8) and two LMSs (LMS1 and LMS2). STUMP8 and LMS2 were primary lesions that had not yet relapsed, while LMS1 was the relapse of STUMP4. The array platform used in this study covers 27,800 CpG Island (CGIs) of the human genome and all the data (percentages and frequencies) are referred to the total number of CGIs included in the array. We calculated the percentages of unmethylated/methylated/undetermined CGIs and we observed that STUMP4, the primary lesion that relapsed, had a divergent methylation pattern compared to the other three samples (Figure 2A, *p* < 0.01 Student’s *t* test). In particular, the percentage of methylated CGIs for all chromosomes in STUMP4 was significantly lower (*p* < 0.01, Chi square test) compared to the one observed in the other three samples, which presented similar percentages, around 50%, of methylated and unmethylated CGIs (Figure 2B).

In order to perform a Gene Ontology (GO) analysis, we set up two lists of genes on the basis of the promoter methylation status. The first list included the 667 genes which were methylated, while the second one the 2220 genes that resulted unmethylated in all samples. Cancer-related GO terms were grouped in different functional categories, as previously described [6]. Each category was scored based on its own percentage of genes belonging to that specific category and normalized to the total number of genes. ‘Metabolism’ and ‘transcription and gene expression’ were the most represented categories in both lists of methylated and unmethylated genes. However, no statistically significant differences were identified (data not shown).

We subsequently refined our analysis, limiting the GOgroups to ‘biological process’ aspects (see Materials and Methods section). The most significant GO-groups included 336 genes (*p*-value = 1.92 × 10^−35^) and 99 genes (*p*-value = 1.68 × 10^−9^) from the unmethylated and methylated gene lists respectively. We found that gene associated GOterms such as ‘transcription and gene expression’, ‘cell cycle’ and ‘cell signalling’ were detected in both the unmethylated and methylated genes’ lists. Conversely, GOterms such as ‘transport’ and ‘DNA repair and chromatin remodeling’ and ‘development and morphogenesis’ and ‘cell death and apoptosis’ were found almost exclusively in the unmethylated or methylated group of genes, respectively.

**Figure 3:**
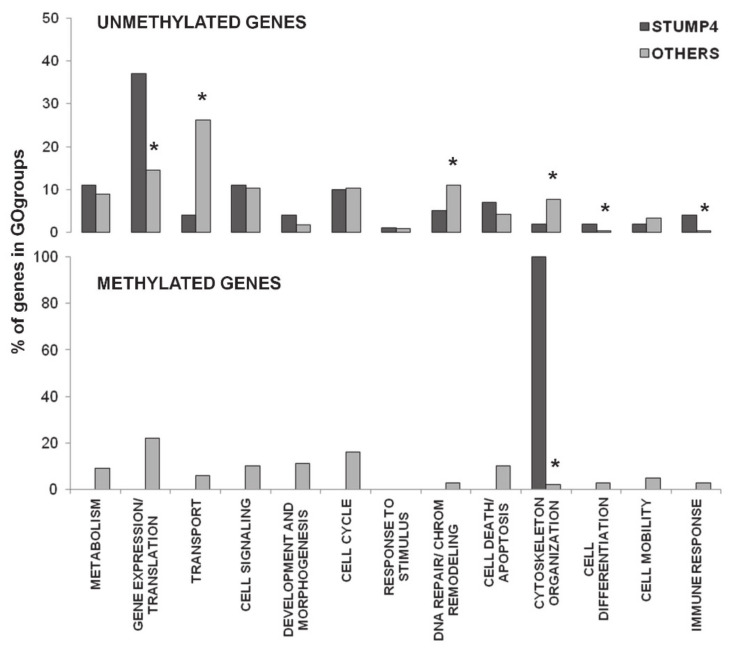
Gene Ontology results. Percentage of unmethylated and methylated genes in GOgroups. * *p* < 0.05.

Given the divergent distribution of CGI methylation percentages in STUMP4, we then compared its methylation pattern to the one of the other three samples. We found that 943 unmethylated genes and 180 methylated genes were exclusive to STUMP4, suggesting a specific methylation signature for this tumor. We restricted the analysis to ‘biological process’ aspects observing that 236 of the unmethylated genes in the most significant GO-group (*p*-value = 2.33 × 10^−11^) were mainly involved in ‘transcription and gene expression’, ‘metabolism’, ‘cell signaling’, ‘cell cycle’ and ‘cell death and apoptosis’ categories, as verified in the previous analysis. Surprisingly, only four of the methylated genes were included in the most significant GO-group (*p*-value = 0.00648) and were involved in ‘cytoskeleton organization’. Furthermore, statistical analysis (chi-square test) revealed a significant difference for some categories between STUMP4 and the other samples (Figure 3).

The analysis’ results allowed to speculate on the potential activated “cell signalling” pathways among the samples. For example, IK beta kinase/NFK beta cascade was mainly represented in STUMP4’s exclusively unmethylated gene list, while the Wnt signalling pathway was detected in the list of genes found to be methylated in all the samples (Table S2).

Afterwards, we focused on the different methylation patterns between the STUMP primary lesion and its recurrence (STUMP4 vs. LMS1). In order to perform the Gene Ontology analysis, we established two lists of genes. The first list included 628 genes, which were found methylated in STUMP4 and unmethylated in LMS1. Conversely, in the second list 2972 genes were reported resultingunmethylated in STUMP4 and methylated in LMS1. As expected, ‘metabolism’ and ‘transcription and gene expression’ were the most represented biological functions in both samples, but no statistically significant differences were identified (data not shown). However, the percentage of unmethylated promoter CGIs in the ‘Cell signalling’ category differed in the two samples: STUMP4 displayed 3.1% of unmethylated genes (92/2972) compared to 9.2% (58/628) of LMS1 (*p* < 0.01, chi square test). In conclusion, we may assume that Ras protein signal transduction, Wnt signalling pathway and NFKbeta cascade were more involved in the primary lesion than in its respective recurrence, being more represented in STUMP4 unmethylated promoters (Table 4).

## 3. Discussion

The World Health Organization classifications indicate that uterine smooth muscle tumors that cannot be histologically diagnosed as unequivocally benign or malignant should be termed STUMP [7]. For this reason, many authors are working to obtain a better definition of diagnostic and prognostic criteria. Several immunohistochemical markers have been analyzed, as well as genetic alterations, but a definitive response has not been obtained yet.

We reported an array-CGH analysis performed on 23 USMTs, including 14 STUMPs, in order to extend the genomic knowledge on this type of tumor. A similar analysis, on a comparable number of STUMPs, has been previously reported [4] identifying a genomic index, based on array-CGH data, as a recurrence predictor. However, we did not find that the genomic index is a recurrence predictor for our patients.

We found that the number of CNAs correlates with primary STUMPs and LMSs prognosis. In fact, samples derived from patients with a worse prognosis displayed a higher number of CNAs compared to the ones derived from patients with a good one.

In order to identify CNAs with a potential prognostic value, we selected the most frequently shared CNAs between STUMP and LMS samples. Array-CGH data showed a high number of gains and a very low number of losses in these lesions. Our hypothesis is that genomic losses would be hidden due to a previous step of genome endoreduplication, as we previously suggested [5]. The presence of polysomy for chromosomes 3, 7 and 17 detected by fluorescence in situ hybridization support this hypothesis (Figure S2).

It is already known that copy number gains or amplifications are associated with protein overexpression in cancer. Eight samples (four STUMPs and four LMSs) shared gain or amplification of *PRKDC* (DNA-Dependent Protein Kinase Catalytic Subunit). This gene is a key component of the non-homologous end joining pathway for DNA repair and its overexpression and/or copy number gain have been observed in several cancer types, such as lung cancer, liver cancer and colorectal cancer, and are associated with more advanced tumor grade and poor survival [8,9]. Recently, its high expression has been associated with poor survival in gastric cancer patients [10] and in both treated and untreated breast cancer patients [11]. In our samples, copy number gain was not associated with poor prognosis; however, it was not identified in leiomyomas. This makes it a very interesting target.

*MLL3* (Mixed Lineage Leukemia 3) copy number gain or amplification was shared among seven samples (three STUMPs and four LMSs). Intriguingly, two STUMPs subsequently relapsed and the third was a relapsed STUMP. Mutations of this gene were reported in different tumors [12], but not in STUMP or leiomyosarcoma.

*ROCK2* and *CTSB* gains or amplifications were shared among six samples (both STUMPs and LMSs). ROCK2 (Rho associated coiled-coil containing protein kinase 2) upregulation has been described in human glioblastoma CSCs [13], in gastric cancer [14], as well as in ovarian cancer samples [15]. Moreover, *ROCK2* expression level has been associated with worse prognosis in osteosarcoma tissues [16]. *CTSB* (cathepsin-B) overexpression has been identified in several tumors with different effects on patient survival [17]. However, high expression of CTSB has been related to poor survival in glioblastoma patients and involved in promoting temozolomide intrinsic resistance [18].

*STAT2* has a well-known role in the anti-viral, immunomodulatory, anti-apoptotic and anti-proliferative effects of IFN-I [19]. Moreover, its high expression in different tumors, such as melanoma, colon adenocarcinoma, breast cancer and lung cancer, has been associated with poor prognosis, highlighting it as a promising therapeutic target [20]. In our samples, its gain/amplification was identified in one relapsing STUMP and its recurrence and in three LMSs, both primary and relapsed.

Interestingly, three out of four relapsing STUMPs shared an amplification of the *PUM2* gene. *PUM2* is an RNA-binding protein involved in embryonic development, cell differentiation and stem cell proliferation. Its role has also been reported in several tumors, such as glioblastoma, where it was overexpressed and promoted cell proliferation [21], or breast tumor, where it promoted the stemness of cancer cells [22].

We refined the analysis comparing only the relapsing STUMPs with LMSs, and we found some shared CNAs. In particular, we observed a copy number gain in four samples (one STUMP and three LMSs) involving *STAG1* (Stromal Antigen 1). Its amplification and overexpression in breast and ovarian cancer cell lines has been previously demonstrated [23].

The same STUMP sample (the only relapsed in a LMS) shared *RFC4* (Replication Factor C Subunit 4) gain with three LMSs. Overexpression of *RFC4* commonly occurs in colorectal cancer and higher levels of RFC4 protein expression correlate with a worse overall survival [24]. Moreover, its overexpression in tumor tissues predicted poor survival in hepatocellular carcinoma and it was also considered a potential therapeutic target [25].

Finally, *ELF2* (E74 Like ETS Transcription Factor 2) copy number gain was shared among only three samples, but its role in tumorigenesis has been well described. In fact, overexpression of *ELF2* enhanced tumor cell proliferation [26] in nasopharyngeal carcinoma, while its silencing had the opposite effect [27].

We previously identified the amplification of *BCL2* gene in primitive STUMPs and their corresponding relapsed tumors [5]. This observation has not been confirmed in this enlarged cohort, where *BCL2* amplification was observed in different samples (eight STUMPs and five LMSs), not only the ones that relapsed. It is therefore clear that an increase of analyzed cases is crucial for the validation of new targets. In this context, we collected and examined 14 STUMPs, a significant number for this rare type of tumor.

Epigenomic modifications, such as DNA methylation, are an integral part of the molecular determinants, contributing to malignancy [28]. For the first time in our knowledge, we mapped out a methylation profile for this type of tumors. Considering the global DNA methylation data, an overall CGI hypomethylation of the STUMP that relapsed was noticed compared to the other three samples. Although the analysis was performed on few cases (two STUMPs and two LMSs), it is interesting that this finding is similar to the one we previously published on three glioma stem cell lines [6]. This analogy could be explained by a greater presence of cancer stem cells in STUMP4, which would justify the worse prognosis with relapse and death of the patient.

In cancer, the role of promoter hypermethylation in silencing tumor suppressor genes and its impact on tumor initiation, progression and prognosis are well known. Even though DNA hypomethylation was the first epigenetic disruption in cancer [29], it took more studies to define its implications in tumorigenesis leading to genomic instability [30], enhanced expression [31], loss of imprinting [32] and abnormal X-chromosome activation [33]. It is interesting to note that the total number of CNAs of STUMP4 is doubled compared to its recurrence and threefold compared to the other STUMP, confirming the association between DNA hypomethylation and genomic instability.

Our data showed a threefold of genes with unmethylated promoter CGIs compared to the methylated ones, considering all four samples. As expected, no statistically significant differences were highlighted by the Gene Ontology analysis comparing the four samples. Conversely, significant differences for several categories emerged from the isolation of the sample with a different profile of methylation (STUMP4), and the comparison with the other three. This analysis highlighted specific signaling pathways that might be preferentially activated in this tumor. The same conclusion emerged thanks to the comparison of the primary sample with its recurrence, outlining a distinctive behavior of the sample with the worst prognosis.

In conclusion, our data demonstrated a more similar genomic profile between STUMPs and LMSs with some genes with a potential prognostic value. Despite the lack of an NGS-based point mutations investigation, which would have given a complete picture of the mutation burden (the landscape), and the number of samples being too small to draw conclusions, the Gene Ontology analysis highlighted some cancer genes that could play a pivotal role in the unexpected aggressive behavior in some of these tumors. These genes could prove to be prognostic markers in the future. Finally, the methylation data appears to be very promising, especially with regards to the divergent profile found in the sample that relapsed, despite the analysis being restricted to only four samples.

## 4. Materials and Methods

### 4.1. Tumor Samples/Patients

A total of 23 uterine smooth muscle tumors: 3 leiomyomas, 14 STUMPs, 5 leiomyosarcomas and 1 undifferentiated sarcoma, were collected from a total of 20 patients (Table 1). Comitato Etico della provincia Monza e Brianza approval and written informed consent was obtained. Patients were selected by a team of pathologists with high level of expertise in the field of soft tissues gynecological neoplasms, supported by a team of gynecologic oncologists with high level of expertise in soft tissue sarcoma.

In our case, we based the diagnosis of STUMP following the WHO indication: a uterine SMT that cannot be diagnosed unequivocally as malignant (at least two of the following criteria: diffuse moderate-to-severe atypia, a mitotic count of at least 10 mitotic figures/10 HPF, tumor cell necrosis, as defined by Bell et al. [34]) or clearly benign.

As far as we are concerned, all the specimens defined as STUMP had <10 mitosis/10 HPFs, but were not classified as benign because of the presence of tumor necrosis even without atypia or additional histological parameters that may predict adverse outcome, as reported by Gupta et al. [35] (briefly: remarkable atypia and borderline mitosis, atypical mitoses, epithelioid differentiation, vascular involvement, infiltrative/irregular margins).

### 4.2. Immunohistochemistry

Immunohistochemical staining was performed on FFPE (4% formalin) sections of 1-µm thickness. The entire pre-treatment process of deparaffinization, rehydration and epitope retrieval was performed using PT LINK (Dako, supplied by Agilent Technologies, Santa Clara, CA, USA). Then the sections were placed into the Autostainer Link 48 with the EnVision FLEX visualization system.

Estrogen receptor (ER), progesterone receptor (PgR), p16, p53, Ki67 (for mitotic activity) and other markers were evaluated (all antibodies were purchased from Dako, except for p16 antibody that was from Gennova Scientific, Sevilla, Spain). For interpretation of immunohistochemical staining, the system proposed by Ip et al. [36] was adopted. In particular, the percentage of positive nuclei for ER, PgR and Ki67 and a scale of positivity (neg: no staining, 1+: <33% of positive cells, 2+: 33–66% of positive cells, 3+: >66% of positive cells) for p16, p53, myogenic markers (actin, desmin) and CD10 were reported in Table 1. For p16, either strong nuclear or cytoplasmic staining or a combination, was considered positive. For p53, only nuclear staining was considered positive.

### 4.3. DNA Extraction from FFPE Tissues

DNA extraction from Formalin-Fixed Paraffin-Embedded (FFPE) tissues was performed by Invisorb^®^ spin Tissue Mini Kit (Stratec Molecular GmbH, D-13125, Berlin, Germany) using 10 µm slices from formaline-fixed paraffin embedded biopsies. All DNAs were quantified by Nanodrop-1000 (ThermoFisher, Wien, Austria) and DNA integrity was measured through agarose gel electrophoresis; generally all FFPE DNA appeared partially fragmented.

### 4.4. Array Comparative Genomic Hybridization (Array-CGH)

Sample preparation, slide hybridization and analysis were performed using SurePrint G3 Human CGH Microarray 8 × 60 K (Agilent Technologies, Santa Clara, CA, USA) based on the UCSC Genome Browser hg18, NCBI build 36.1, March 2006 with 41Kb overall median probe spacing (33 Kb in RefSeq genes), according to the manufacturer’s instructions for FFPE samples. A control female DNA from a healthy female blood donor was used as sex-match reference. This control DNA was validated by testing on CGH microarray using as references available commercial European Male DNA NA-1289 and European Female DNA NA-12878 (Coriell Institute, Camden, NJ, USA) and by self-hybridization. All the samples were heat fragmented at 99 °C for 10 to 20 min and 250 ng of fragmented DNA per sample were directly labelled by Genomic DNA ULS-labelling Kit (Agilent Technologies) according to the protocol. ULS-labelling kit does not copy or amplify the input DNA, so the yield after the labelling is the same as the input DNA. The arrays were scanned at 2 µm resolution using the Agilent microarray scanner and analyzed using Feature Extraction v10.7 and Agilent Genomic Workbench v5.0 softwares (Agilent Technologies, Santa Clara, CA, USA). The Aberration Detection Method 2 (ADM-2) algorithm was used to compute and assist the identification of aberrations in a given sample (threshold = 5.0), assigning a statistical score based on the average quality weighted log ratio (DLRS) of the sample and reference channels. ADM2 threshold filter selected at least three contiguous probes with an average absolute value of the log2ratio change of 0.60 across the aberration in order to define gains and losses. Additional abnormalities were manually noted when detected via visual inspection of the experimental data points for each chromosome. The estimated percentage of mosaicism was calculated using the formula determined by Cheung SW et al. [37].

### 4.5. MeDIP-Chip

Methylated DNA immunoprecipitation and chip hybridization were performed following the guidelines of Agilent Microarray Analysis of Methylated DNA Immunoprecipitation Protocol (Version 1.0, Agilent Technologies, Santa Clara, CA, USA) as previously described [6]. Briefly, purified genomic DNA was sonicated to obtain fragments of 200–600 bp in size and 5 µg of sheared DNA was immunoprecipitated using 50 µl of pan-mouse IgG Dynal magnetic beads (Life Technologies Italia, Monza, Italy) and 5 µg of 5-methylcytosine antibody (Eurogenetec, Seraing, Belgium). MeDIPed DNA and reference DNA were purified and directly labelled with Cyanine 5- and Cyanine 3-dUTP nucleotides, respectively, using the SureTag DNA Labelling Kit (Agilent Technologies, Santa Clara, CA, USA). Cy5- and Cy3-labelled samples were combined in a single mixture and hybridized onto a Human CpG Island Microarray 1 × 244 K (Agilent Technologies, Santa Clara, CA, USA) for 40 h at 67 °C. Microarrays were scanned at 5 µm using an Agilent microarray scanner and images analyzed with the Agilent Feature Extraction software v10.7. Data were analyzed by means of the Agilent Genomic Workbench v5.0 software (Agilent Technologies, Santa Clara, CA, USA). The full list of CpG islands (CGIs) analyzed is based on the UCSC Genome Browser hg18, NCBI build 36.1, March 2006. Data were further analyzed according to the methodological approach conceived by Dr. Ravid Straussman and colleagues in 2009 [38]. Briefly, probe z-scores for each CGI were averaged to obtain the Island Methylation Score (IMS) and IMS distribution allowed threshold setting for determining the methylation status of each CGI. CGIs resulted fully methylated (+1) or fully unmethylated (−1) or undetermined (0). Undetermined CGIs were not considered for Gene Ontology analysis.

### 4.6. Bionformatic Analysis

The Gene Ontology analysis was performed using the GOstat software (http://gostat.wehi.edu.au/ accessed on 21 May 2020 [39]) in order to identify the possible enrichment of functional groups related to “biological process”, in a specific input list of genes. GOterms were divided in cancer-relevant functional categories as previously described [6]. Analyzed categories were: ‘cell cycle’, ‘cell death and apoptosis’, ‘response to stimulus’, ‘cytoskeleton organization’, ‘cell signalling’, ‘development and morphogenesis’, ‘cell differentiation’, ‘immune response’, ‘cell motility’, ‘metabolism’, ‘transcription and gene expression’, ‘intracellular transport’, ‘DNA repair and chromatin remodeling’.

GOstat analysis was also performed narrowing the GO hierarchy through the use of ‘biological_function’ as a keyword.

## Figures and Tables

**Table 1:** Clinical features.

Patient	Sample	Histotype	Age at Diagnosis	Surgery	Mitosis	KI-67	Markers	ER	PgR	p16	p53
1	LM1	Leiomyoma	28	n.a.	0	5%	n.a.	n.a.	n.a.	n.a.	n.a.
2	LM2	Leiomyoma	52	vaginal histerectomy, bilateral salpingo oohorectomy	<1 × 10 HPF	0	n.a.	n.a.	n.a.	n.a.	n.a.
3	LM3	Leiomyoma	40	n.a.	0	<5%	n.a.	n.a.	n.a.	n.a.	n.a.
4	STUMP1	STUMP	56	n.a.	2 × 10 HPF	10%	Actin 3+	neg.	neg.	neg.	2+
	STUMP2	STUMP	59	n.a.	5 × 10 HPF	10%	Actin 3+	neg.	neg.	neg.	3+
5	STUMP3	STUMP	47	n.a.	4–5 × 10 HPF	<1%	Actin 2+	neg.	neg.	neg.	neg.
	US1	Undiff. sarcoma	48	total abdominal histerectomy, bilateral salpingo oophorectomy	14 × 10 HPF	20%	Actin r.e.	neg.	neg.	neg.	3+
6	STUMP4	STUMP	41	Myomectomy	3 × 10 HPF	<5%	Actin 1+, Desmin 3+, CD10 1+	30%	50%	neg.	2+
	LMS1	Leiomyosarcoma	45	n.a.	15 × 10 HPF	40%	Actin 1+, Desmin 3+, CD10 1+	neg.	60%	neg.	neg.
7	STUMP5	STUMP	26	n.a.	6-7 × 10 HPF	30%	Actin 1+	neg.	neg.	neg.	neg.
	n.a.	Leiomyosarcoma	n.a.	n.a.	n.a.	n.a.	n.a.	n.a.	n.a.	n.a.	n.a.
8	n.a.	STUMP	47	n.a.	n.a.	n.a.	n.a.	n.a.	n.a.	n.a.	n.a.
	STUMP6	STUMP	54	total abdominal histerectomy, bilateral salpingo oophorectomy	2 × 10 HPF	3%	Actin 3+, Desmin 3+, CD10 neg.	90%	70%	neg.	2+
9	n.a.	STUMP	60		n.a.	n.a.	n.a.	n.a.	n.a.	n.a.	n.a.
	STUMP7	STUMP	65	total abdominal histerectomy, bilateral salpingo oophorectomy	5–6 × 10 HPF	n.a.	Actin 3+, Desmin 1+, CD10 neg.	50%	neg.	neg.	neg.
10	STUMP8	STUMP	40	Myomectomy	9 × 10 HPF	30%	Actin 1+	neg.	20%	neg.	3+
11	STUMP9	STUMP	31	n.a.	1 × 50 HPF	<1%	Actin neg., Desmin 2+	neg.	70%	neg.	neg.
12	STUMP10	STUMP	30	Myomectomy	4–6 × 10 HPF	<1%	Actin 2+	10%	70%	neg.	neg.
13	STUMP11	STUMP	42	total abdominal histetectomy, left salpingo oophorectomy	1–2 × 10 HPF	<1%	Actin neg.	neg.	40%	neg.	r.e.
14	STUMP12	STUMP	47	total abdominal histerectomy, bilateral salpingo oophorectomy	4–5 × 10 HPF	<5%	Actin 1+	neg.	neg.	neg.	neg.
15	STUMP13	STUMP	58	total abdominal histerectomy, bilateral salpingo oophorectomy	1–2 × 10 HPF	<1%	Actin 1+	neg.	50%	neg.	neg.
16	STUMP14	STUMP	34	laparothomy	0–1 × 10 HPF	1%	Actin 3+, Desmin 3+	30%	80%	neg.	neg.
17	LMS2	Leiomyosarcoma	49	total abdominal histerectomy, bilateral salpingo oophorectomy	9 × 10 HPF	30%	Actin 2+, Desmin 2+	20%	90%	neg.	neg.
18	LMS3	Leiomyosarcoma	63	n.a.	15 × 10 HPF	10%	Actin 1+	neg.	neg.	neg.	3+
19	LMS4	Leiomyosarcoma	50	laparoscopic histerectomy, bilateral adnexectomy	3–4 × 10 HPF	10%	Actin 1+, Desmin 1+	neg.	30%	neg.	r.e.
	n.a.	Leiomyosarcoma	54	n.a.	n.a.	n.a.	n.a.	n.a.	n.a.	n.a.	n.a.
20	n.a.	Leiomyosarcoma	68	n.a.	n.a.	n.a.	n.a.	n.a.	n.a.	n.a.	n.a.
	LMS5	Leiomyosarcoma	73	n.a.	1 × 10 HPF	<1%	Actin 1+	40%	60%	r.e.	r.e.
	n.a.	Leiomyosarcoma	77	n.a.	n.a.	n.a.	n.a.	n.a.	n.a.	n.a.	n.a.

ER: estrogen receptor, HPF: high power field, LM: leiomyoma, LMS: leiomyosarcoma, n.a.: not available, neg: negative immunohistochemistry, PgR: progesterone receptor, r.e.: rare elements, STUMP: smooth muscle tumors of uncertain malignant potential, US: undifferentiated sarcoma (more information in the Materials and Methods section).

**Table 2:** Array-CGH results.

Patient	Sample	Follow Up	OS	CNAs		
				Total	Gain	Loss
1	LM1	NED	6	110	106	4
2	LM2	NED	12	123	114	9
3	LM3	n.a.	2	268	239	29
4	STUMP1	n.a.	5	25	23	2
	STUMP2			17	17	0
5	STUMP3	DOD	2	365	342	23
	US1			28	28	0
6	STUMP4	DOD	7	256	238	18
	LMS1			126	121	5
7	STUMP5	DOD	1	58	58	0
	n.a.					
8	n.a.	ALIVE	13			
	STUMP6			116	108	8
9	n.a.	NED	8			
	STUMP7			94	88	6
10	STUMP8	NED	14	88	80	8
11	STUMP9	ALIVE	9	82	81	1
12	STUMP10	n.a.	10	40	38	2
13	STUMP11	NED	18	97	97	0
14	STUMP12	NED	10	156	155	1
15	STUMP13	NED	16	85	81	4
16	STUMP14	NED	7	59	58	1
17	LMS2	ALIVE	8	204	194	10
18	LMS3	n.a.	n.a	199	189	10
19	LMS4	DOD	7	262	234	28
	n.a.					
20	n.a.	AWD				
	LMS5		23	166	148	18
	n.a.					

LM: leiomyoma, STUMP: smooth muscle tumors of uncertain malignant potential, LMS: leiomyosarcoma, US: undifferentiated sarcoma, NED: no evidence of disease, AWT: alive with tumor, DOD: dead of disease, OS: overall survival (years from the first tumor).

**Table 3:** Top nine CNAs shared between STUMPs and leiomyosarcomas (all copy number gains).

	*PRKDC*	*MLL3*	*ROCK2*	*CTSB*	*STAT2*	*STAG1*	*RFC4*	*CCRN4L, ELF2*	*PUM2*	*BCL2*
STUMP2	+									
STUMP3	+	+	+	+					+	+
STUMP4		+			+	+	+	+	+	+
STUMP5									+	
STUMP6				+						+
STUMP7	+	+		+						
STUMP9			+							
STUMP10										+
STUMP11			+	+						+
STUMP12	+		+							+
STUMP13			+							+
LMS1	+	+			+					+
LMS2	+			+		+	+			+
LMS3	+	+	+		+		+	+		+
LMS4		+			+	+	+			+
LMS5	+	+		+	+	+		+		+

**Table 4:** Cell signaling Pathways with the related genes in STUMP4 and its recurrence LMS1.

Cell Signaling Pathway	Genes with a Significant GOterm	Unmethylated Genes in STUMP4 (Methylated in LMS1)	Methylated Genes in STUMP4 (Unmethylated in LMS1)
**NFKβ cascade**	22	*NDFIP1, MAP3K7IP2, PLK2, NDFIP2, NLRC3, PPM1A, SECTM1, TRIM13, IRAK1, TSPAN6, VAPA, TNFAIP3, BIRK2, C9orf89, EDG2, GOLT1B, LTBR, PRDX3, TNFRSF1A, RPS6KB2*	*ZDHHC13, TFG*
**WNT receptor signaling pathway**	16	*CSNK2B, LRRFIP2, PYGO2, PORCN, DKK1, FZD3, FZD6, FZD8, HBP1, WNT10A, WNT2B, WNT6, WNT7A, DIXDC1, TLE4, WNT11*	/
**Smoothened signaling pathway (Hedgehog signaling pathway)**	1	*CTNNA1*	/
**Negative regulation of WNT pathway**	1	*FRZB*	/
**Regulation ARF GTPase activity (Ras protein transduction)**	24	*ARGEF2, PSD3, CENTB2, CENTD1, GNB1, DOK3, EVI5, PSD4, RASSF1, RASSF6, GBF1, DDEF1, DOK2, SMAP1, PSCD1, PSCD4, ARFGEF1, CTGLF4*	*ARFGAP1, FBXO8, IQSEC1, TBC1D22A, TBC1D15, TBC1D16*
**Ras GTPase activity**	15	*TBC1D5, TBC1D8, TBC1D9, TBC1D9B, TBC1D2B, TBC1D4, EVI5L, SGSM1, TBC1D10A, TBC1D20, TBC1D25, TBC1D10C, TBC1D12, CENTG1, RABGAP1*	/

## Data Availability

Data available on request due to restrictions eg privacy or ethical.

## Supplementary Materials

The following are available online at https://www.mdpi.com/1422-0067/22/4/1580/s1, Figure S1: Array-CGH results, Figure S2: Fluorescence in situ hybridization results, Table S1: CNAs shared between STUMPs and LMSs, or STUMPs and LMs, Table S2: Methylation status of gene involved in cell signaling pathway.

## Abbreviations

CGH	Comparative Genomic Hybridization
CGIs	CpG Island
CNAs	Copy Number Alterations
GO	Gene Ontology
LM	Leiomyoma
LMS	Leiomyosarcoma
STUMPs	Uterine Smooth muscle Tumors of Uncertain Malignant Potential
USMTs	Uterine Smooth Muscle Tumors
