# Supplementary material for: Genomic and Epigenomic Profile of Uterine Smooth Muscle Tumors of Uncertain Malignant Potential (STUMPs) Revealed Similarities and Differences with Leiomyomas and Leiomyosarcomas
Source: Int J Mol Sci. 2021 Feb 4;22(4):1580. doi: 10.3390/ijms22041580 (PMC7914585; doi:10.3390/ijms22041580)
Supplement: Supplementary file 1 [file ijms-22-01580-s001.zip › Supplementary figures.docx]

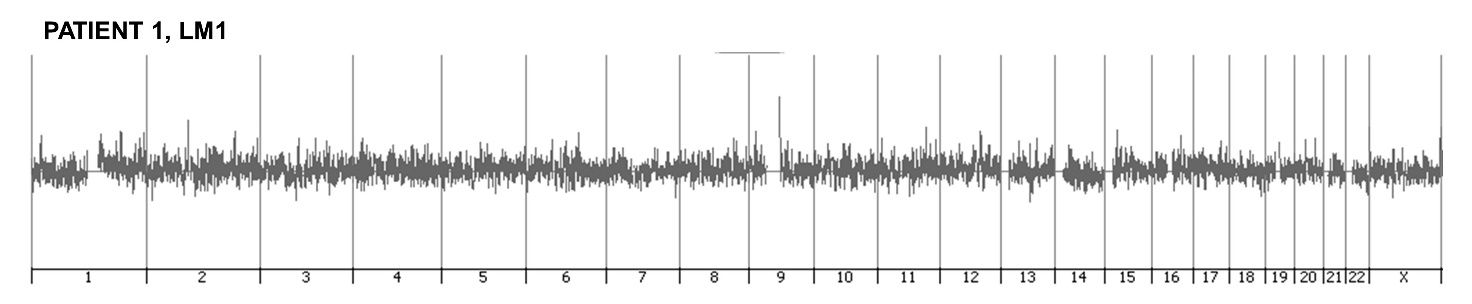

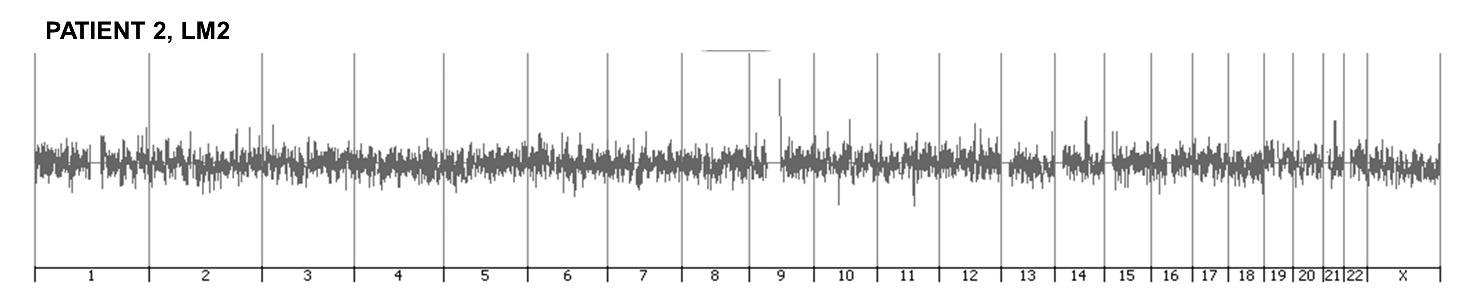

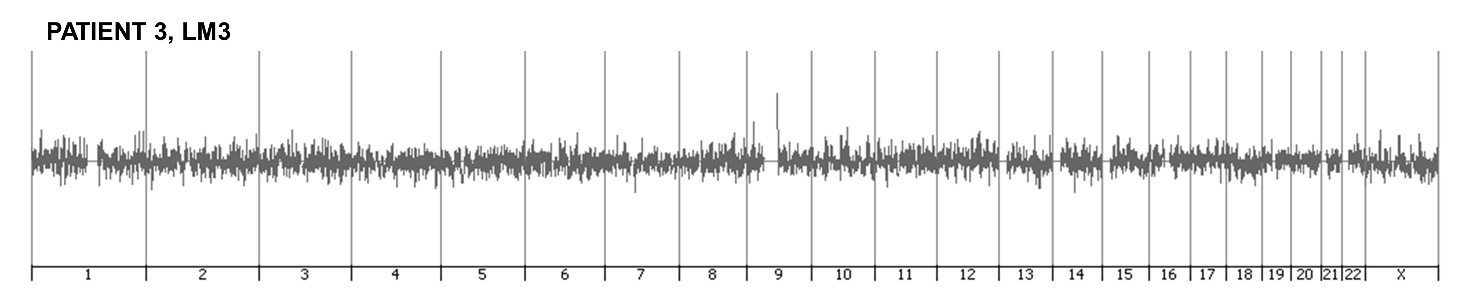

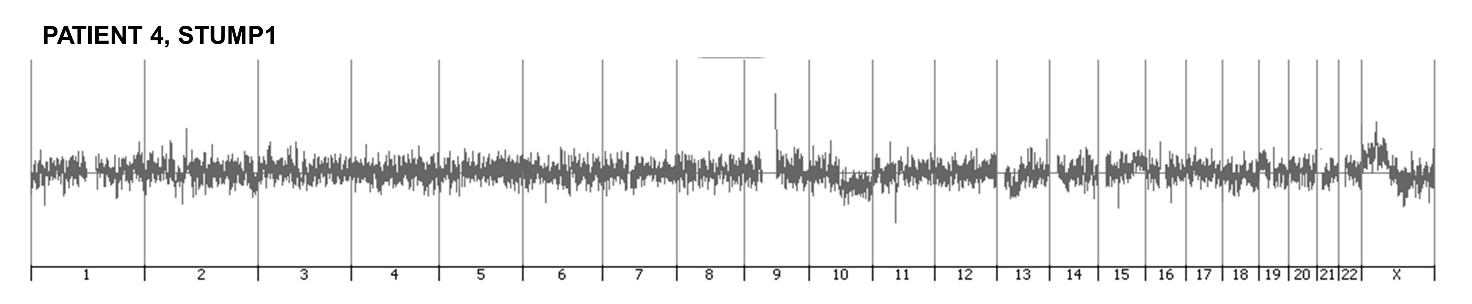

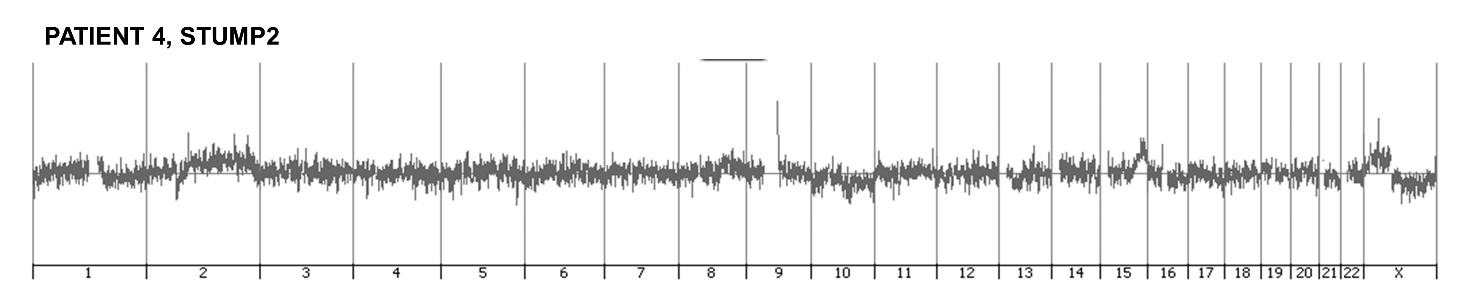

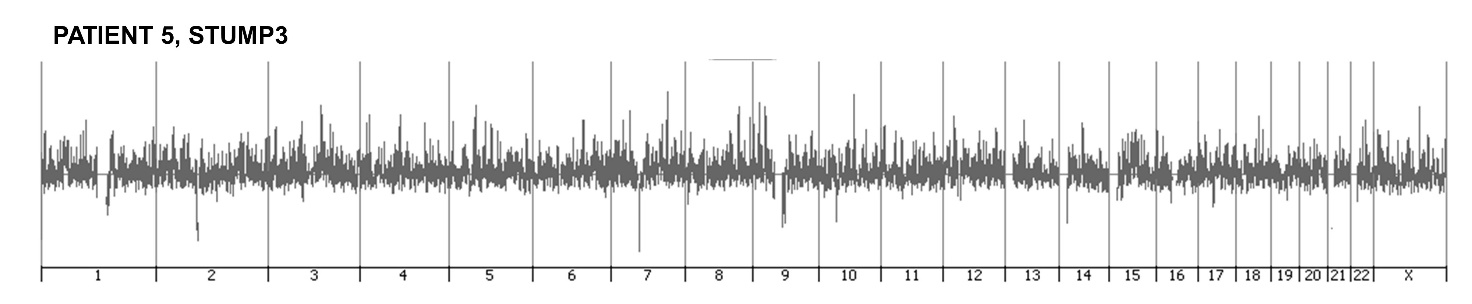

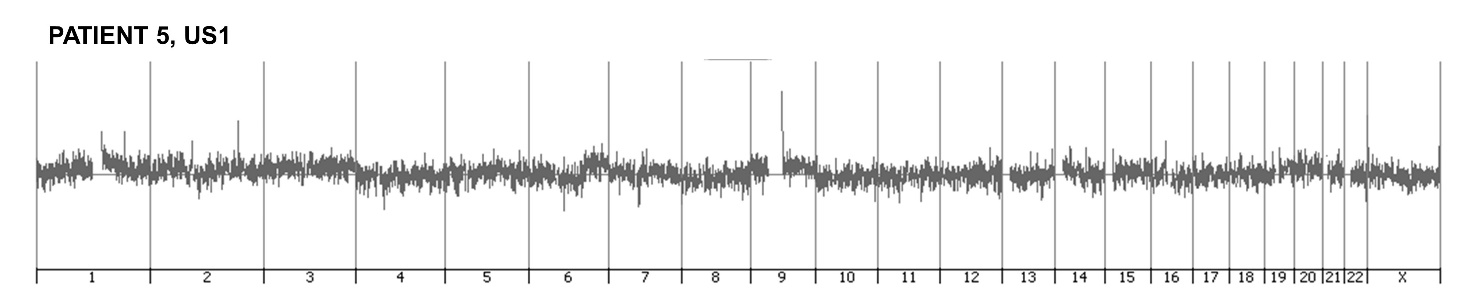

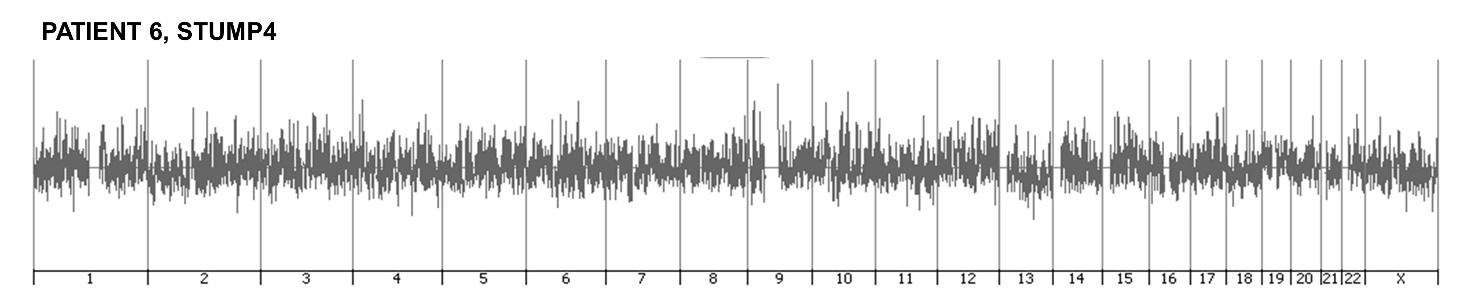

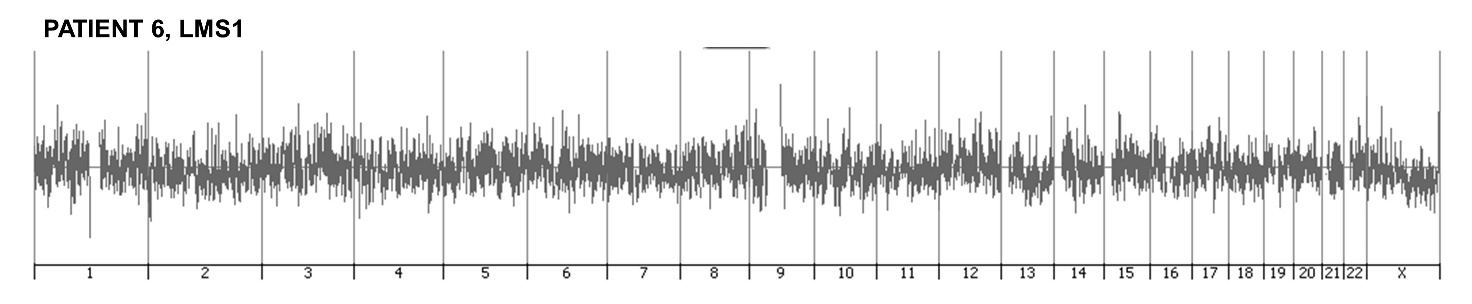

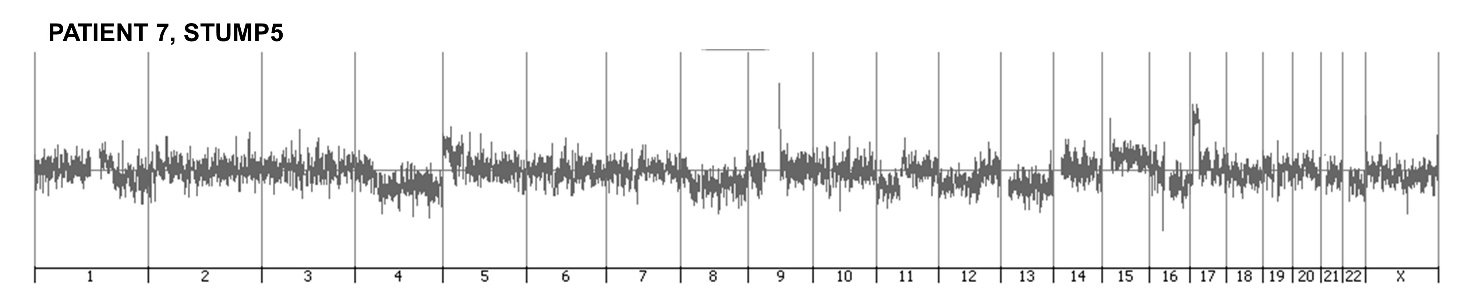

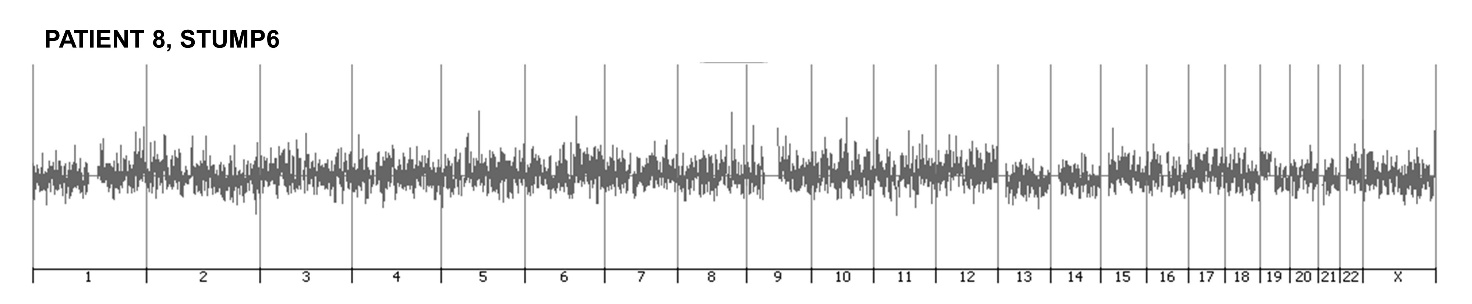

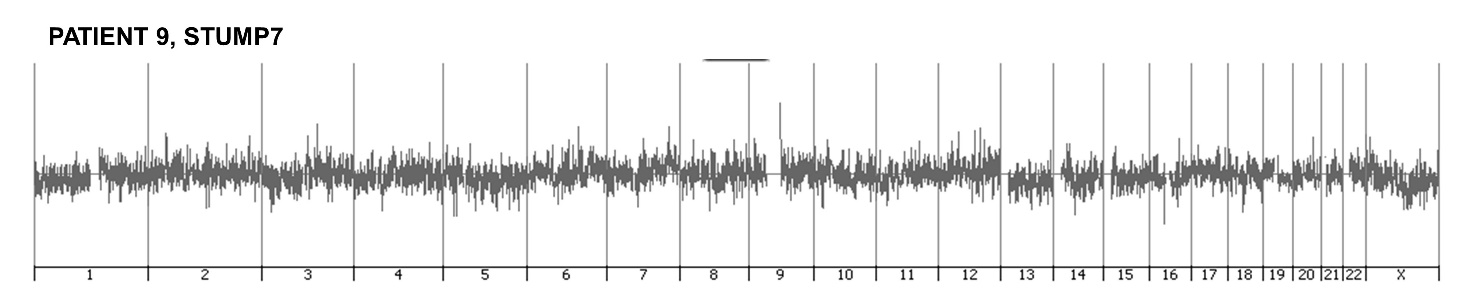

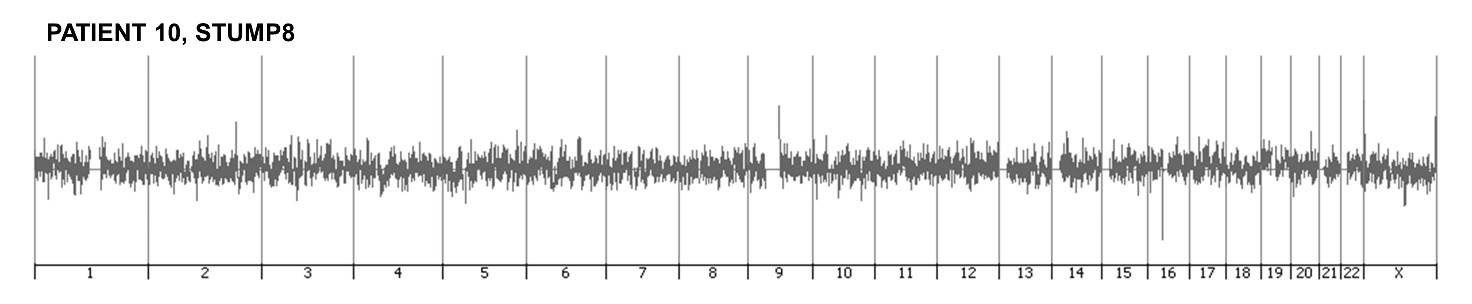

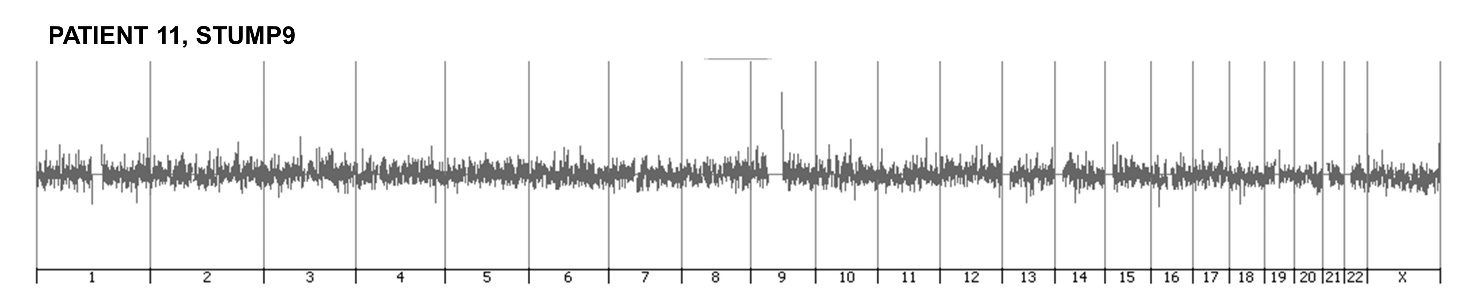

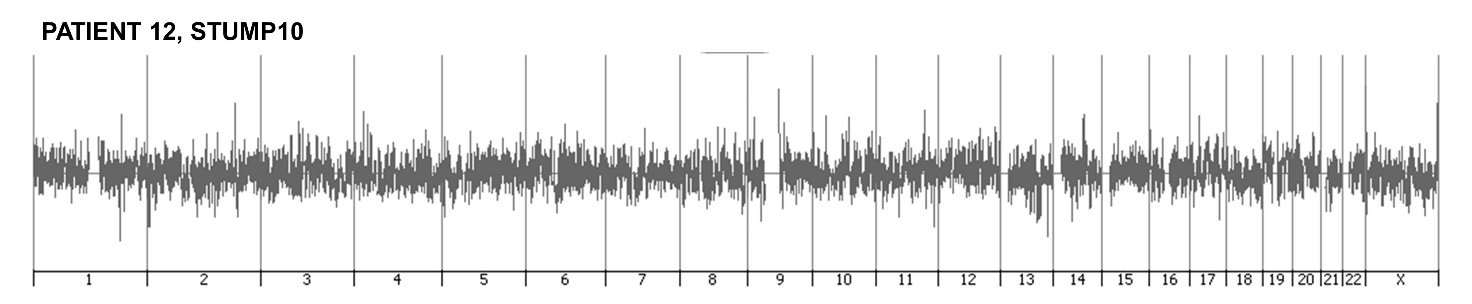

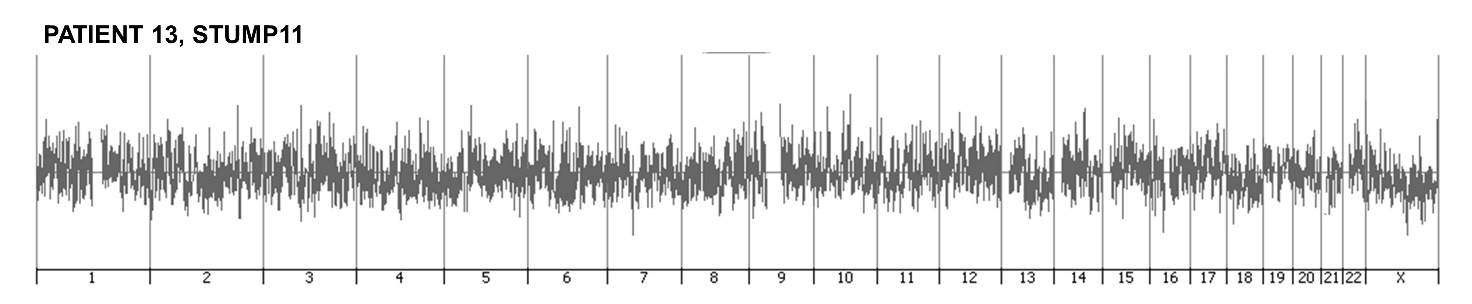

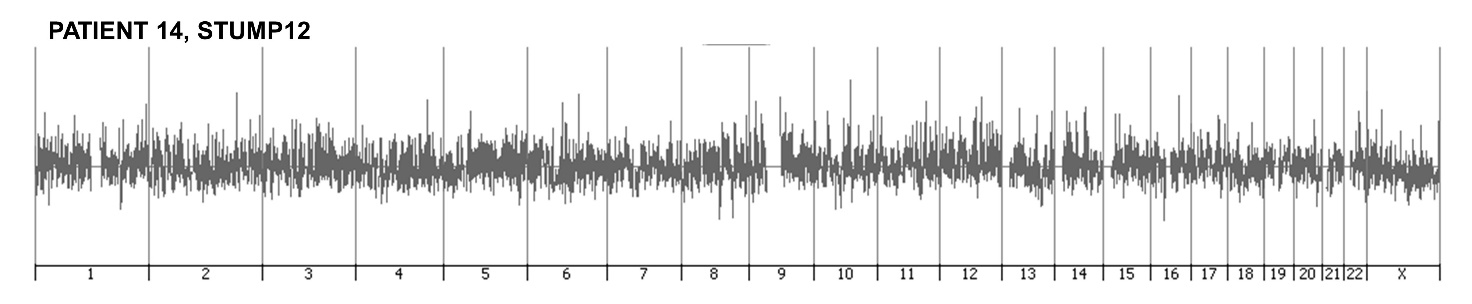

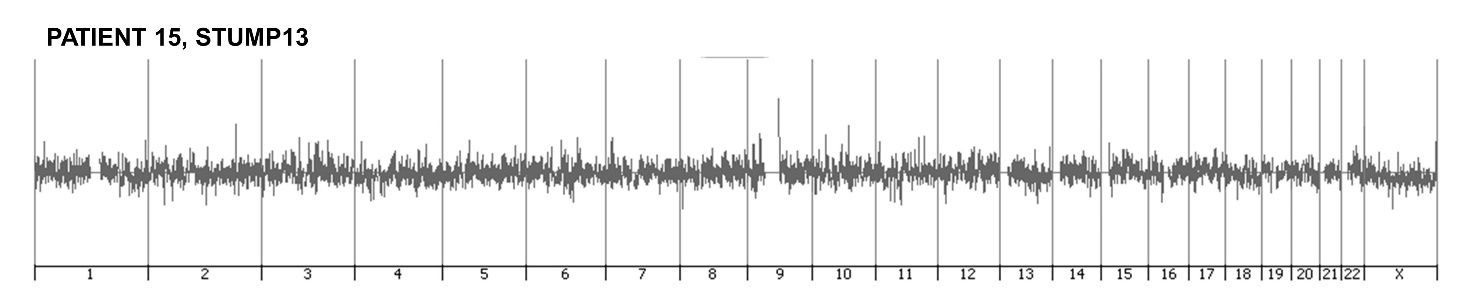

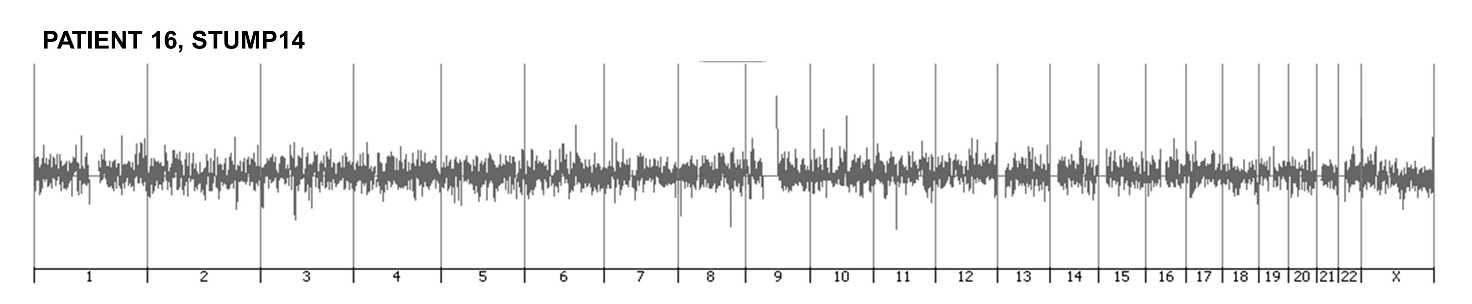

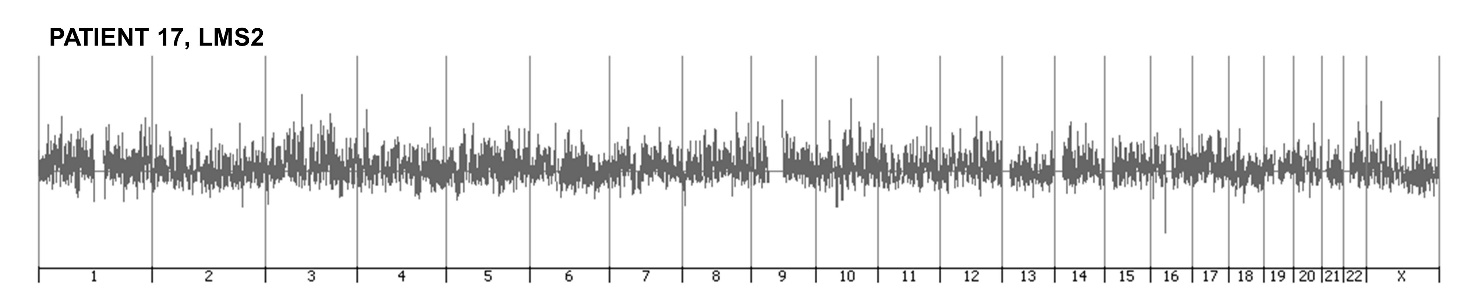

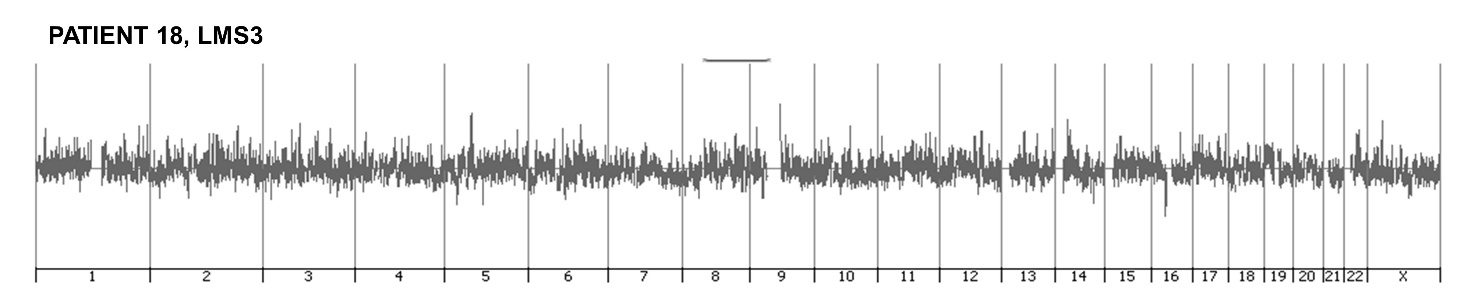

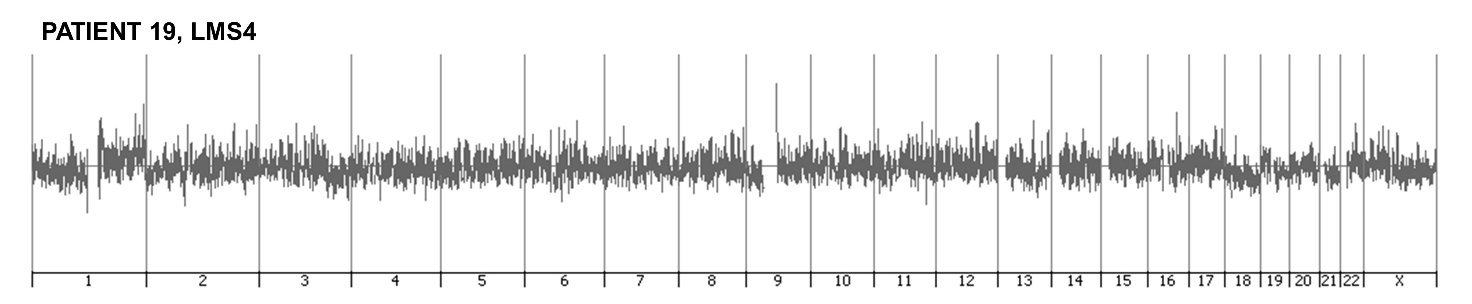

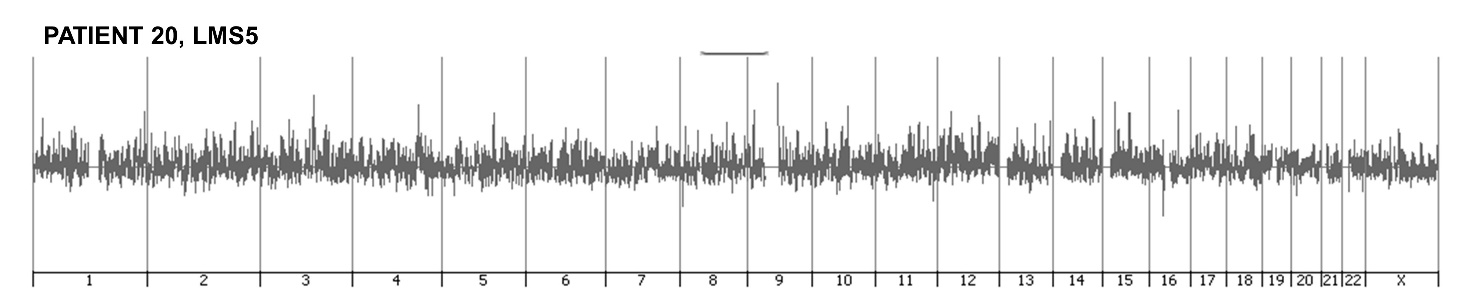


**Figure S1. Array-CGH results.** CNAs’ distribution by chromosome for each patient. The plots’ order follows the table 1.


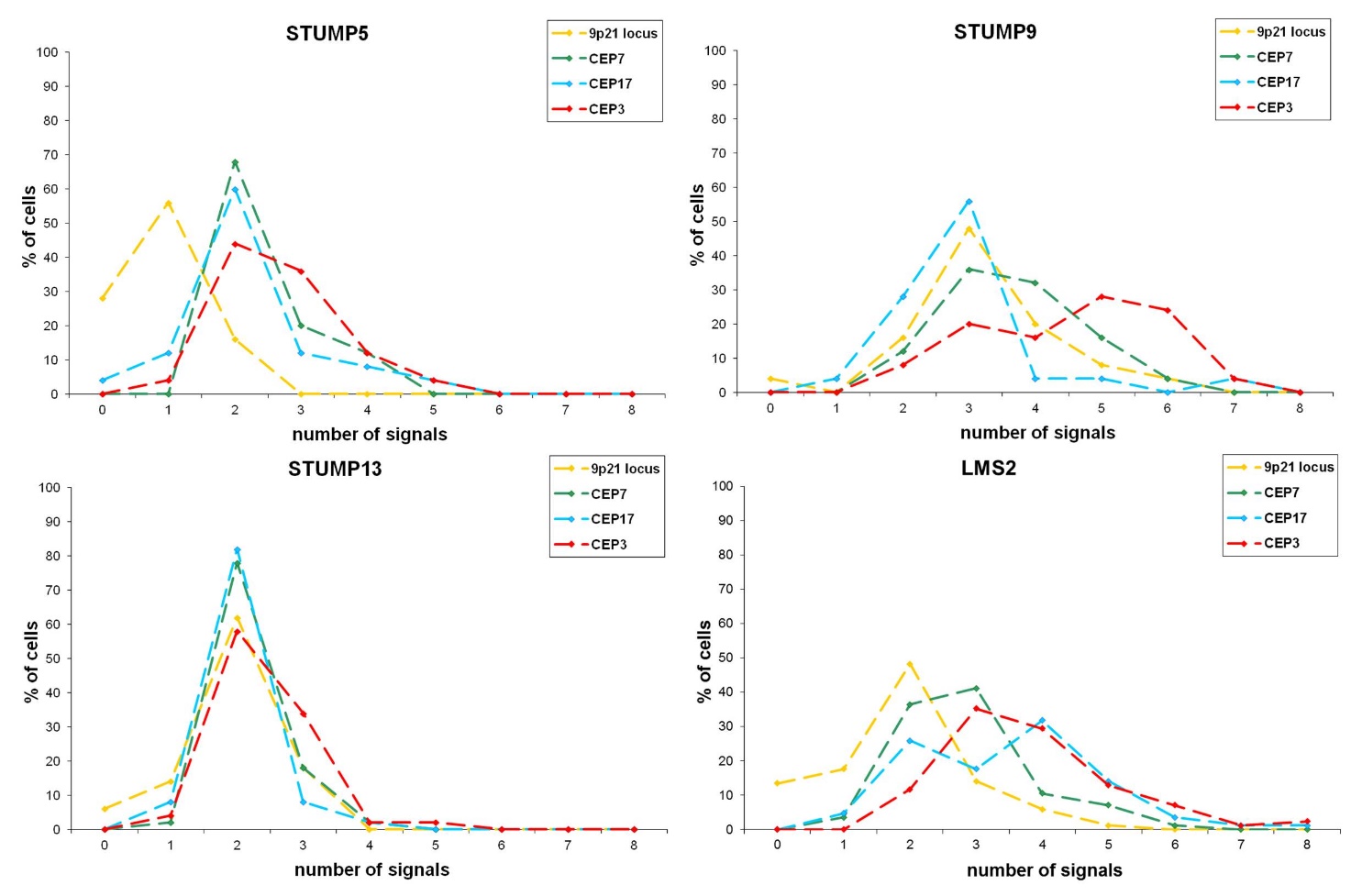


**Figure S2.** **Fluorescence in situ hybridization results.** Distribution of UroVysion probe signals on 3 STUMP samples and 1 leiomyosarcoma. CEP: centromeric probe; CEP7: centromeric probe of chromosome 7 (green); CEP17: centromeric probe of chromosome 17 (aqua); CEP3: centromeric probe of chromosome 3 (red); 9p21 locus specific probe (yellow). Fluorescence in situ hybridization analysis of FFPE tissue sections was performed using UroVysion bladder cancer kit (Vysis, Abbott Park, IL, USA) according to the manufacturer’s instructions. At least 25 cells were analysed for each sample. All digital images were captured using a Leitz microscope (Leica DM 5000B, Leica Microsystems GmbH, Leica Microsystems, Milan, Italy) equipped with a charge-coupled device (CCD) camera and analyzed by means of Chromowin software (Tesi Imaging, Milano, Italy).
