# Supplementary material for: Genomic and Epigenomic Profile of Uterine Smooth Muscle Tumors of Uncertain Malignant Potential (STUMPs) Revealed Similarities and Differences with Leiomyomas and Leiomyosarcomas
Source: Int J Mol Sci. 2021 Feb 4;22(4):1580. doi: 10.3390/ijms22041580 (PMC7914585; doi:10.3390/ijms22041580)
Supplement: Supplementary file 1 [file ijms-22-01580-s001.zip › Table S2.doc]

| **STUMP4 vs ALL** | |
| --- | --- |
| **BEST GOgroup p-value=2.33e-11** | |
| **GENE INVOLVED IN CELL SIGNALING: UNMETHYLATED IN STUMP4 (METHYLATED IN OTHERS)** | |
| **GENE** | **CELL SIGNALING PATHWAY** |
| *GRIK1* | glutamate signaling pathway |
| *NLRC3* | **IK beta kinase/NFK beta cascade** |
| *EVI5* | regulation of Rab protein signal transduction |
| *IKBKG* | T cell receptor signaling pathway |
| *TGFB3* | positive regulation of MAP kinase activity |
| *PTPLAD1* | **I-kappaB kinase/NF-kappaB cascade** |
| *RASGEF1B* | regulation of small GTPase mediated signal transduction |
| *TSPAN6* | **positive regulation of I-kappaB kinase/NF-kappaB cascade** |
| *NDFIP2* | **positive regulation of I-kappaB kinase/NF-kappaB cascade** |
| *FLT4* | vascular endothelial growth factor receptor signaling pathway |
| *GALR1* | G-protein signaling, coupled to cAMP nucleotide second messenger, regulation of adenylate cyclase activity |
| *SLC9A2* | cell-cell signaling; synaptic transmission |
| *CDC42BPG* | regulation of small GTPase mediated signal transduction |
| *ACVR2A* | transmembrane receptor protein serine/threonine kinase signaling pathway; positive regulation of activin receptor signaling pathway |
| *CD81* | positive regulation of MAP kinase activity |
| *CERK* | G-protein signaling, coupled to IP3 second messenger (phospholipase C activating); protein kinase C activation |
| *VAV3* | regulation of small GTPase mediated signal transduction; regulation of Ras protein signal transduction; regulation of Rho protein signal transduction |
| *RXFP3* | G-protein coupled receptor protein signaling pathway; elevation of cytosolic calcium ion concentration during G-protein signaling, coupled to IP3 second messenger (phospholipase C activating) |
| *TBC1D2B* | regulation of Ras GTPase activity; regulation of Rab GTPase activity |
| *PYDC1* | **negative regulation of transferase inhibition of NF-kappaB transcription factor; positive regulation of cytokine secretion** |
| *C9ORF89* | **negative regulation of I-kappaB kinase/NF-kappaB cascade** |
| *MED4* | steroid hormone receptor signaling pathway, androgen receptor signaling pathway |
| *GALR3* | G-protein signaling, coupled to cAMP nucleotide second messenger, regulation of adenylate cyclase activity; negative regulation of adenylate cyclase activity |
| *TMOD2* | positive regulation of G-protein coupled receptor protein signaling pathway |
| *ITGA7* | integrin-mediated signaling pathway |
| *SOCS2* | JAK-STAT cascade |

| **STUMP4 vs ALL** | |
| --- | --- |
| **BEST GOgroup p-value=1.68e-09** | |
| **GENE INVOLVED IN CELL SIGNALING: METHYLATED IN ALL THE SAMPLES** | |
| **GENE** | **CELL SIGNALING PATHWAY** |
| *ADRB1* | G-protein signaling, coupled to cyclic nucleotide second messenger; G-protein signaling, coupled to cAMP nucleotide second messenger; regulation of adenylate cyclase activity; positive regulation of adenylate cyclase activity |
| *PIK3CG* | second-messenger-mediated signaling; phosphoinositide-mediated signaling |
| *FZD5* | **establishment of tissue polarity; Wnt receptor signaling pathway** |
| *CYP26B1* | retinoic acid receptor signaling pathway |
| *IL11* | cell-cell signaling; negative regulation of hormone secretion |
| *GPR98* | cell surface receptor linked signal transduction |
| *MYCBPAP* | cell-cell signaling |
| *AXIN1* | **Wnt receptor signaling pathway through beta-catenin** |
| *WIF1* | **Wnt receptor signaling pathway** |
| *WNT8B* | **Wnt receptor signaling pathway, calcium modulating pathway** |
